# Research on Optimization of Process Parameters of Traditional Chinese Medicine Based on Data Mining Technology

**DOI:** 10.1155/2022/2278416

**Published:** 2022-03-02

**Authors:** Xue Li, Hao Yue, Jinlong Yin, Yan Song, Jinling Yin, Xinlei Zhu, Bingchang Huang

**Affiliations:** ^1^Jilin Ginseng Academy, Changchun University of Chinese Medicine, Jilin, Changchun 130117, China; ^2^Department of Food Science and Engineering,Jilin Business and Technology College, Jilin, Changchun 130062, China; ^3^Department of Clinical Medicine, Changchun University of Chinese Medicine, Jilin, Changchun 130117, China; ^4^Jilin Haotai Health Industry Development Co., Ltd, Jilin, Changchun 130041, China

## Abstract

Data mining technology and methods are used to effectively optimize manufacturing process parameters due to the complexity and uniqueness of the process parameters. The data-mining-based optimization method for traditional Chinese medicine (TCM) process parameters is presented, along with a list of process parameters that have shown to be effective in actual production. The influencing factors of process parameters are analyzed and modeled using an attribute weight analysis and classification analysis algorithm. The optimization scheme of process parameters that meet the requirements is selected, and an example is given for verification, by selecting data records that fall within a certain error range and incorporating the rules of association knowledge discovery. The support vector classification algorithm has a higher accuracy, despite the algorithm's results being understandable. The support vector regression algorithm developed a reliable process optimization model.

## 1. Introduction

Traditional Chinese medicine (TCM) is the treasure of the Chinese nation. TCM-manufacturing industry is a sunrise industry with international competitiveness and independent intellectual property rights in China's pharmaceutical industry [1]. The active ingredient extract of TCM has various uses and can be used in drugs, health products, and cosmetics. Therefore, the extraction of effective components of TCM has a wide range of practical scope and application prospects [2]. However, at present, the technical level of China's TCM-manufacturing industry has become increasingly backward compared with foreign countries. It is urgent to use modern science and tech to transform the traditional manufacturing mode of TCM products, promote the modern production of TCM, and then pave the way for TCM to rush out of the country and go to the world [3]. TCM production has moved from digitization to intelligence as a result of the rise of intelligent manufacturing technology around the world. It is necessary to continuously optimize the production process in the process of intelligent TCM production in order to gradually improve the product quality index [4]. One of the important contents of the modernization of TCM manufacturing is extracting stable active components from medicinal materials and ensuring the uniformity and stability of the drug dose-response relationship. As a result, TCM extraction is a critical step in the TCM-manufacturing process [5, 6]. It is the process of a solvent entering a medicinal material and transferring the active ingredients from the solid to the liquid phase. The choice of process parameters, such as extraction temperature, extraction time, and solvent volume, has a significant impact on the leaching of effective components in medicinal materials during the extraction process. Currently, orthogonal design is used to optimize the extraction process parameters [7].

With the promotion of advanced production technology, the manufacturing of TCM has gradually tended to be standardized. However, at present, the intelligent production of TCM has just started. It is a typical interdisciplinary subject, which needs the cooperation of control theory, pharmaceutical theory, and computer theory [8]. In the production of TCM, the selection of process parameters has an important impact on production efficiency and economic benefits. The selection of process parameters generally depends on workers' experience. These parameter values are only applicable to specific TCM products. When the production conditions change, the product quality is difficult to guarantee [9]. In the production of TCM, the production process needs to be continuously optimized to ensure the stability of the production process and the reliability of product quality. At present, the optimization of production process is still in the primary stage. The common method is to design comparative experiments in the laboratory environment to obtain the optimal production process scheme and then move the last to the production environment. In order to complete the comparative experiment, it often requires huge human and material resources [10]. TCM contains a variety of effective components, and different effective components have different effects. The extract can be used in medicine, health care products, and cosmetics. Therefore, the extraction of effective components of TCM has a wide range of practical value and application prospects. In this paper, data mining technology is applied to the optimization of process parameters of TCM, and the optimization model of process parameters of TCM is established.

Large-scale production data can be obtained as a result of the development of intelligent TCM manufacturing, and these actual production data have more practical significance than laboratory experimental data [11]. It contains the harsh conditions that occur during mass production. Using production data to create a production process model and optimize it through data mining rather than design and comparative experiment is not only a future trend but also a pressing problem to solve [12]. In the production of modern TCM, computers collect a large amount of data. Manually understanding, identifying, and optimizing these data and their relationships is typically difficult. There is no reference experience or manual for a new TCM product in the face of a new process, only accumulated data, especially for a new TCM product. At this time, it is necessary to quickly establish optimized process parameters according to traditional technologies and methods. The difficulty is great. As the manufacturing system of Chinese patent medicine is too complex and the front and rear sections interact with each other, at present, the TCM production still adopts the end-point detection standard of production quality index for quality control, which means that the inspection of the effective components of finished drugs is the only standard to judge whether the drugs are qualified [13]. In the actual machining process of flexible manufacturing system, there are often many factors affecting the results. It is difficult to give an accurate mathematical model by using the traditional reasoning method. Through in-depth study of industrial process optimization, this paper puts forward an intelligent optimization scheme of TCM production process. Decision tree algorithm and support vector classification algorithm are used to construct the classifier of extraction times. Support vector regression algorithm is used to establish regression prediction models for extraction time and solvent amount, respectively. The experimental results show that the error of the optimal process parameters obtained by this algorithm is small, the results are accurate and reliable, and it has practical value, which can provide some reference for the industrialization of the extraction of effective components of TCM.

## 2. Related Work

Zhang et al. [14] used association rules to investigate the drug use law of ancient and modern asthma prescriptions, compare ancient and modern asthma drugs, and identify the core drugs for asthma treatment. Liu et al. [15] collected data from qualitative and quantitative angles to explore the syndrome differentiation of kidney yang deficiency syndrome based on the scale of kidney yang deficiency syndrome. A qualitative score was used to calculate the occurrence frequency of syndrome differentiation factors, and a quantitative score was used to perform hierarchical cluster analysis. Chen et al. [16] discussed the stroke cases of ancient famous doctors using data mining technology to analyze association rules, obtain the TCM commonly used by ancient famous doctors in the treatment of stroke, and identify common medicinal materials for stroke treatment. Data samples must be manually measured off-line in the research field of ultrasonic extraction of effective components of TCM, according to Hoogeveen et al.'s study [17]. This method of data collection has a long cycle and is extremely difficult. As a result, the amount of sample data available is limited, which is a small sample problem. Chaovalitwongse et al. [18] believe that although there are many medical record databases, the simple database has only query function and loses the core analysis function of the database. Bandaru et al. [19] emphasize the application of data mining technology in TCM medical case sorting and give the process of data mining technology in medical case information mining and the data mining method applied to medical case sorting. Lee et al. [20] propose to use data warehouse and data mining technology to save the clinical diagnosis and treatment data of old experts, analyze and mine the collected data using association rule analysis, factor analysis, discriminant analysis, and decision tree and form a knowledge base. Statistical analysis software is a data mining tool, which uses descriptive statistics analysis, frequency analysis, correlation analysis, discriminant analysis, decision tree, and neural network to analyze and mine the data related to syndrome differentiation in the database [21]. This paper explores the dialectical thinking mode of famous TCM in the diagnosis and treatment of vertigo and makes a preliminary summary of the law. Asadi et al. [22] proposed a set of relatively perfect time-series modeling theory and analysis method. In the process of optimizing the process parameters of coal plant sorting, Hyunjin and Eunok [23] designed a process-prediction real-time feedback system, which can detect the process parameters on the line in real time, predict the sorting index in real time, provide feedback and guide the production, and finally achieve the optimal sorting effect. Guo and Zhou [24] proposed data mining on medical records of threatened abortion, summarized the treatment characteristics and academic ideas of famous doctors in treating specific diseases, and played a guiding role in clinical diagnosis and treatment of diseases. When optimizing the high-pressure water jet cutting process, Angeli et al. [25] and Yd et al. established a polynomial regression model using the process parameters and cutting depth of the cutting process and optimized the parameters in the cutting process through the regression model. However, the ultrasonic extraction mechanism is complex, and the optimal process parameters are difficult to determine. Therefore, the information technology method can be combined with the ultrasonic extraction process. Introducing data mining technology into the study of modernization of TCM is another new attempt of applying computer technology to the study of modernization of TCM. This paper proposes an intelligent optimization solution of production process based on data mining, uses production data to establish an accurate quality index prediction model, screens key process parameters, and then optimizes key process parameters based on the above two steps. Thus, the intelligent optimization of process parameters using historical data is realized. The optimized process parameters are used for actual production, and the process parameters are monitored in real time. The results show that the model can optimize the process parameters and achieve good results in practical application.

## 3. Methodology

### 3.1. Data Mining

Data mining is a very dynamic research direction in the field of artificial intelligence [27, 28] and database, including classification, clustering, regression, association rule discovery, and other mining tasks. Data mining is a process of automatically searching for hidden and special relational information from a large amount of data. For massive and disorderly data, comprehensive analysis based on individual thinking will be limited by cognitive level, thinking mode, subjective factors, research methods, and research scope. It is an important part of the research on modernization of Chinese medicine to use data mining technology to acquire knowledge, remove the false and preserve the true, remove the rough and extract the fine from numerous Chinese medicine resources, so as to promote the development of Chinese medicine.

For complex and multidimensional systems, data mining technology is appropriate. We can find the rules using corresponding algorithms and break through the limitation of “information is jumbled and knowledge is scarce” in TCM with the help of a large amount of data. The primary function of data mining is discovery or prediction, and it encompasses a wide range of disciplines and methods. After “discovery,” find out the hidden information patterns in a large amount of data, or find out the things or special cases in the designated area that deviate from the normal situation [29]. Prediction is the process of predicting the future based on patterns discovered. It is currently a very active research area in the fields of artificial intelligence and databases, with mining tasks such as classification, clustering, regression, and association rule discovery being included.

For the continuous storage and multilevel mining of TCM data, a high-quality and long-term updating of the data mining platform is essential. In the age of Big Data, creating a multimethod collaborative Chinese medicine mining model can help with TCM inheritance and development. Finding valuable hidden knowledge in large databases is the main problem that data mining must solve. We can create a systematic model and provide it to relevant departments for reference by analyzing and summarizing this valuable information. The medical field frequently employs drug frequency analysis, association rules, factor analysis, cluster analysis, and neural network analysis. Each of these algorithms has its own set of characteristics, and different algorithms can be used for different topics.

### 3.2. Application of Data Mining Technology in Optimizing Extraction Process of TCM

**Figure 1:**
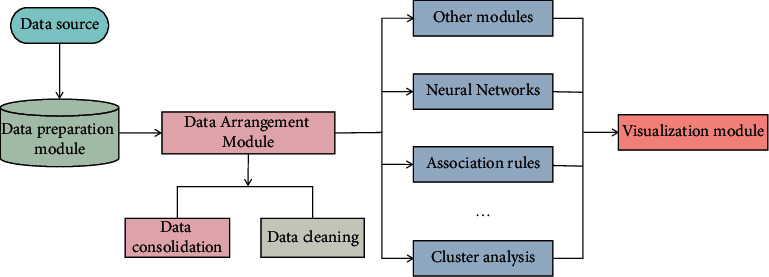
The functional structure diagram of the data mining system for Chinese medicine production.

TCM prescription is the main means of TCM treatment. Through legislation based on differentiation of symptoms and signs, prescription is unified by law, and medicine is sent by prescription. In the prescription, there is an intricate correspondence among prescription, medicine, and syndrome [30]. As data mining technology can reflect the mutual mapping relationship between multidimensional data, it provides a very powerful research tool for the research of modern prescriptions. Under the premise of the trend of Big Data in the whole medical industry, at present, there is no system as a research system of TCM to be popularized and used in various famous doctor research systems. Only by accumulating data can we develop more knowledge to guide clinical or prescription research, which is more conducive to systematic experience inheritance of famous doctors. China's medical industry is currently in the primary stage of Big Data, which brings opportunities to the development of TCM. Through the platform of Big Data, there are more researches focusing on the origin of academic theory of TCM. A common problem is that the interpretation of data mining is not detailed enough. Using data mining to dig out potential and meaningful rules from the database can only be transformed into knowledge through high-quality data interpretation and analysis. The system established in this paper consists of four main modules: data preparation, data sorting, data analysis, and data analysis result visualization module. The main functional modules of the system are shown in Figure 1.

At present, in the field of TCM, data mining is most widely used in the research of TCM, and some progress has been made. In the process of extraction, the changes of temperature, density, pressure, liquid inlet, and other parameters have an impact on the quality of semifinished products. Using the data mining algorithm for scientific data analysis, the process parameters closest to the production results and the data interval of the process parameters that can produce qualified semifinished products can be obtained. Data mining can find the relationship between syndrome types and symptoms through a large number of clinical data, so as to assist clinical diagnosis. It has certain reference value for clinical treatment of diseases to obtain the commonly used drugs, pairs of drugs, and core prescriptions for the treatment of diseases through data mining, but it has the disadvantage of not ensuring the authenticity, accuracy, and universality of the data.

Medical records are complicated records of Chinese medicine diagnosis and treatment activities. We can master more and more objective syndrome rules if the factors are standardized first, and then data mining technology is used to analyze the contribution of qualitative variables to diseases and syndromes. The study should focus on the quality of screening documents, obtain first-hand case data from the original authors, further investigate the etiology and pathogenesis, improve clinical practice and observe the curative effect, or conduct animal experiments to further verify the effect and mechanism. The characteristics of data in TCM are discrete, continuous, mixed, and so on. The preprocessing of these data is extremely difficult, and the mining process necessitates frequent human-computer interaction, necessitating the use of professional technicians at every stage. We can only dig out truly valuable knowledge by choosing reasonable digging methods for different problems under the guidance of TCM theory and closely combined with clinical practice.

### 3.3. Optimization of Process Parameters of TCM Based on Data Mining

**Figure 2:**
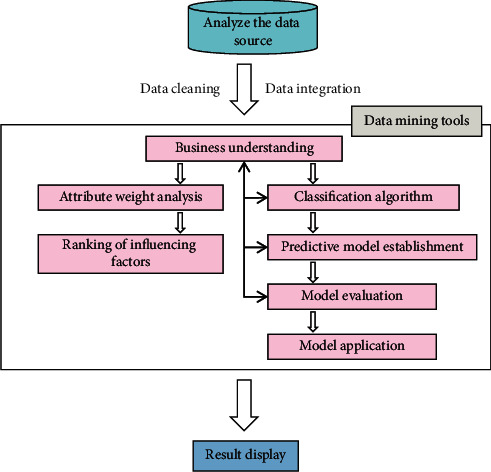
Data mining model for optimization of process parameters.

In the research of TCM, there are a lot of qualitative descriptions and a lot of vague concepts about the description of drugs, diagnosis and treatment process and disease symptoms. Especially for the description of drugs, the phenomenon that one drug has the same name as different drugs is also very common. The prescription of TCM takes the disease as the main body, adopts legislation based on syndrome differentiation, unifies prescription by law and sends medicine by prescription, and is determined by clinical practice. As data mining technology can reflect the mutual mapping relationship between multidimensional data, it provides a very powerful research tool for the research of modern prescriptions. In this paper, through the seamless integration of database and data mining algorithm, a process parameter optimization model based on data mining is constructed, which mainly uses data mining method to analyze and predict the extraction data of TCM. The data mining model of process parameter optimization is shown in Figure 2.

This system includes the function of adding multiple data files for analysis. Users do not need to combine multiple files into one by hand or third-party software to prepare data. The data related to the production of TCM are stored in different files according to the date. When importing these files, they are merged into a complete data set according to the records, and the data are cleaned to ensure that more accurate information can be mined.

The basic idea of support vector machines (SVMs) is dimension upgrading and linearization. Through the kernel function, the sample space is mapped to a high-dimensional or even infinite-dimensional feature space, the optimal linear hyperplane is found in the feature space, and the solution of the optimal hyperplane of the SVM is reduced to solving a convex optimization problem, so that the global optimal solution. SVM includes support vector classification (SVC) and support vector regression (SVR).

Let the sample set be AAA. For the linearly separable case, the sample set is separated by the hyperplane *wx* + *b* = 0. The problem of constructing the optimal hyperplane is transformed into

(1) $$\begin{array}{c} \min\varphi \left(w\right)=\frac{1}{2}{\left\|w\right\|}^{2}\\ =\frac{1}{2}\left(w^{T}w\right),\\ s.t.\;y_{i}\left(x_{i}\cdot w+b\right)\geq 1,\;i=1,\ldots ,n. \end{array}$$

Then, the original problem can be transformed into the dual problem of convex quadratic programming as follows:

(2) $$\begin{array}{c} \max\sum_{i=1}^{n}a_{i}-\frac{1}{2}\sum_{i=1}^{n}\sum_{j=1}^{n}a_{i}a_{j}y_{i}y_{j}\left(x_{i}^{T}x_{j}\right),\\ s.t.\sum_{i=1}^{n}a_{i}y_{i}=0,\;a_{i}\geq 0,\;i=1,\ldots ,n. \end{array}$$

According to the Kuhn–Tucker condition, this optimal solution must also satisfy:

(3) $$\begin{array}{c} a_{i}\left[y_{i}\left(w^{T}x_{i}+b\right)-1\right]=0. \end{array}$$

In the case of linear inseparability, non-negative relaxation variables can be introduced. The specific solution method is similar to the case of linear separability. Data mining analysis function includes three functional modules: cluster analysis, association rules, and neural network. By analyzing the characteristics of data that enterprises need to cluster, we know that all data attributes are continuous attributes, and the demand of enterprises is to try to divide the data with similar values into a group, so the best grouping method is to group the data with similar values according to the Manhattan distance between the data.

The multiobjective problem is transformed into a single-objective problem by linear fusion method, and then the single-objective problem is solved. Assuming that the number of process parameters to be optimized is *N*_*v*_, the number of optimized objective functions is *N*_0_, and the objective function is *f*, the problem can be described as follows:

(4) $$\begin{array}{c} \max F\left(x\right)=\max\left(f_{1},f_{2}\ldots ,f_{N_{o}}\right). \end{array}$$

Among them, the following constraints *l* and *h*, respectively, represent the threshold range in the parameter.

(5) $$\begin{array}{c} X=\left\{x_{1},x_{2}\ldots ,x_{N_{v}}|l\leq x\leq h\right\}. \end{array}$$

Usually, the optimal global objective function is constructed by multiobjective fusion, and the multiobjective problem is transformed into a single-objective problem. The general fusion methods include: (1) linear fusion. Linear fusion is the sum operation of each objective function according to a certain proportion, as shown in the following formula.

(6) $$\begin{array}{c} F\left(x\right)=\beta_{0}+\beta_{1}f_{1}+\beta_{2}f_{2}+\ldots +\beta_{n}f_{n}. \end{array}$$

(2) Logical integration: Logic fusion means that the output of the previous objective function is used as the input of another objective function as shown in the following formula.

(7) $$\begin{array}{c} F\left(x\right)=f_{n}\left(f_{n-1}\left(\ldots f_{2}\left(f_{1}\right)\right)\right). \end{array}$$

Regardless of which model fusion method is used, the goal is to reduce a multiobjective function to a single-objective function, which can then be solved using constraints. In the preparation process, there are many process parameter points to investigate, and each process parameter has a different unit of measurement. Even though some parameters have the same unit of measurement, their measured values vary widely, sometimes dramatically. If the collected data are directly analyzed and modeled based on their actual values, the role of some parameters with larger dimensions will be exaggerated, and the actual change relationship between the parameters will be obscured, resulting in the so-called false variation. As a result, the original data must be processed before being analyzed in order to eliminate the influence of dimensional effects of various variables on the model.

Many studies are unable to use randomized controlled trials due to the influence of traditional medical treatment modes and evaluation methods, and their clinical efficacy evaluation is limited to some extent, and the efficacy evaluation system is not perfect, making their research results difficult to be internationally recognized. The majority of data mining analyses are based on disease differentiation and syndrome differentiation laws, such as drug frequency analysis, association rule analysis, and cluster analysis and are combined with current disease understanding. Association rules can be used to analyze the patterns or rules of prescription compatibility. We can analyze the results of correlation data mining among different attributes such as drugs and symptoms, drugs and pathogenesis, and symptoms and pathogenesis using the frequent set method.

## 4. Results Analysis and Discussion

**Figure 3:**
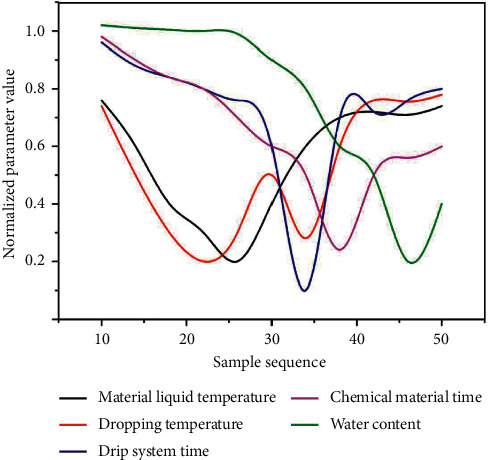
Changes in the main variables.

The neural network module uses the derived association rule module to integrate data, and its attributes include extracting the mean value of liquid inflow, extracting the variance of liquid inflow, extracting the mean value of temperature, extracting the variance of temperature and blending the liquid-solid content. When using attribute weight analysis algorithm and classification analysis algorithm to analyze the data, it is necessary to discretize the surface roughness in the sample data. The main technological parameters affecting the water content of TCM include the water content of extract, the temperature of feed liquid, the dripping humidity, the dripping time, the melting time, and so on. Their changes are shown in Figure 3.

**Figure 4:**
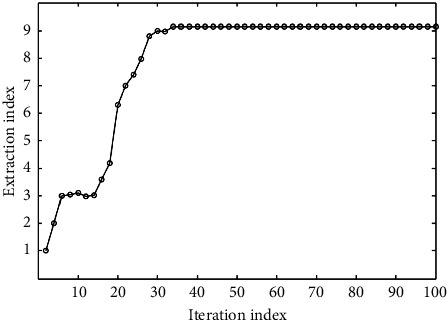
Forecast model optimization.

From Figure 1, it can be seen that there are many uncertain factors in the process of dropping the preparation because the parameters affecting the water content change irregularly. Through the prediction of water content, the value range of each process parameter can be further determined, or the value range of some process parameters can be reduced, so that the production quality can be controlled. The optimization process of process parameters based on the prediction model in this paper is shown in Figure 4. To obtain the optimum conditions for ultrasonic extraction of Shaoyao Gancao Decoction.

The process parameters are modified, and the process parameters are tested in parallel for three times, with the average value taken, according to the aforementioned method, which takes into account the limitations of actual operation. The application of data mining in the field of TCM is dependent on the development of a data platform, and the continuous storage and multilevel mining of TCM data requires a high-quality and long-term updating of the data mining platform. To better serve the inheritance and development of Chinese medicine in the era of Big Data, a good data mining platform must have powerful data collection, management, and analysis functions, as well as the ability to establish a multimethod collaborative Chinese medicine mining model. The association rule module's data discretization system automatically discretizes data using a program that only requires the user to specify the number of divided intervals. When discretizing, it is necessary to keep track of the symbols for different attributes and intervals, as well as the attribute names and marks, which adds to the system's complexity but makes it easier to use.

**Figure 5:**
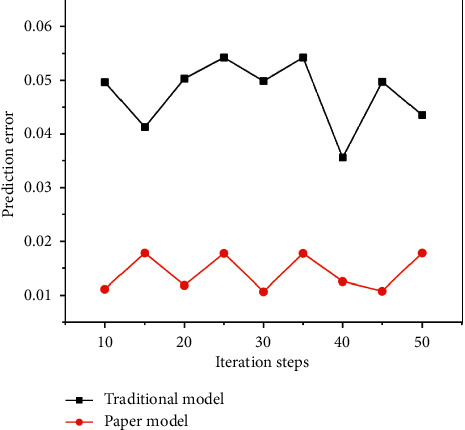
Forecast error analysis.

Fingerprint of TCM is characterized by quantification, specificity, reproducibility and reproducibility, stability, integrity, and fuzziness of detail processing. Through data mining of TCM fingerprint, we can extract the hidden and potentially useful and ultimately understandable information. Taking the collected samples as the test set, the prediction model is simulated and analyzed, and the comparison results of prediction errors of different methods are shown in Figure 5.

**Figure 6:**
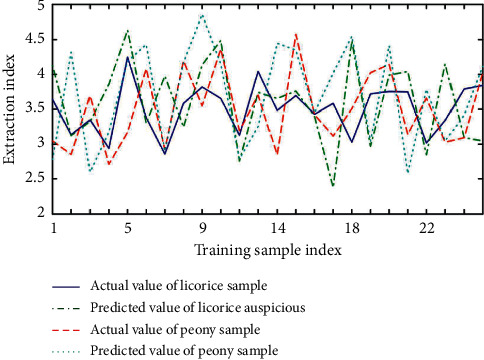
Predictive model training results.

**Figure 7:**
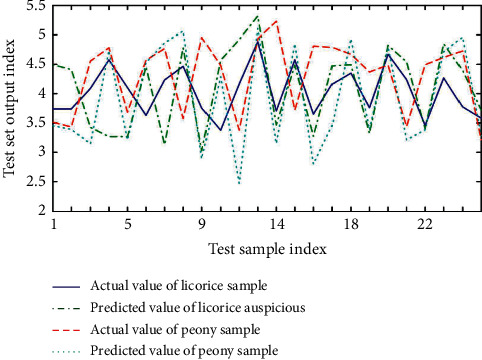
Predictive model test results.

**Figure 8:**
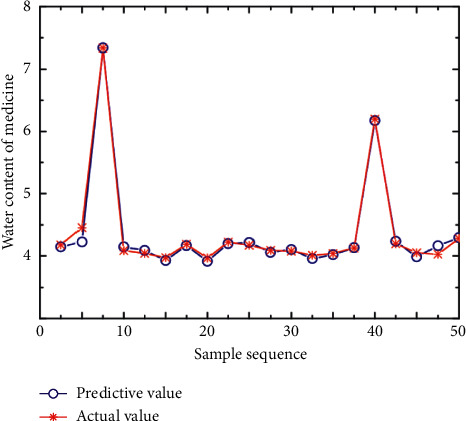
Comparison of predicted and true values.

Generally, neural network is a specific analysis of specific problems. There is no fixed model for all problems. The design of this system has a wide range of applications. It does not mean that a neural network structure is suitable for many practical problems, but users can set the network structure by themselves. Configure the input layer, output layer, and intermediate nodes according to different actual problems, so as to achieve the purpose of predicting results. In order to determine the relationship between process parameters and solid content, we select each attribute in the data except solid content as the input node, including: extracting the mean value of the liquid volume, extracting the variance of the liquid volume, extracting the average temperature, and extracting the four attributes of the temperature variance. The system predicts the solid content based on the information of these several production processes. The optimized input and output parameter values are converted into maximum and minimum inverse transformations, and the process parameter values of the preparation process can be obtained. Based on basic principles and training sample sets, a prediction model was established for the ultrasonic extraction of licorice and peony process parameters. The training result is shown in Figure 6, the test result is shown in Figure 7, and the test error is shown in Figure 8.

Figure 6 shows that the prediction model's training effect is good. Figures 7 and 8 show that the difference between the predicted and actual values of the test sample obtained by the prediction model is generally small, indicating that this model's prediction accuracy is high. Process design knowledge is at the heart of all data in process parameter optimization. This model allows you to mine and analyze any type of continuous data. The importance of influencing factors is determined by establishing the association model of data mining and using attribute weight analysis, and the classification model of data mining is used to predict the cutting parameter scheme, and the scheme is optimized based on the predicted results. The system can analyze quality analysis data and TCM extraction data while also analyzing the data mining function of various data in various fields. It has the advantages of flexible deployment, free configuration, and expandability, and it better solves the problems of TCM extraction production data quality inspection standard optimization and process parameter optimization.

With the development of the times, the number of TCM information resources is increasing rapidly, but the characteristics of TCM information resources are complex and disorderly, which has formed a serious contradiction with the demand for information of professional and technical personnel. To solve this contradiction, an important way is to process all kinds of information and establish a relatively perfect information resource database. The algorithm of data mining part consists of attribute weight analysis algorithm and classification algorithm, and the classification model is built by data mining tools to predict the results. Show the results to users through visualization technology. In this paper, a data mining system platform is built to realize data mining of process parameter data and quality inspection data. Using data mining technology, the relationship between the extraction parameters and the description of the characteristics of TCM was discovered from the extraction data of TCM. The discovered knowledge can guide technologists to scientifically select the influencing factors of orthogonal test and the variation range of each factor level when determining the extraction process of a new drug, so as to ensure that the orthogonal test can obtain reliable optimized results.

## 5. Conclusion

In the research of modernization of TCM, the advanced information processing technology, represented by data mining, is used to study the knowledge acquirement of its implied essence. Systematizing and standardizing the documents left over from past dynasties can further extract and excavate the hidden essence. The research on knowledge acquisition with data mining technology has driven the improvement of the academic level of TCM. In this paper, an intelligent optimization scheme of TCM production process is proposed. From the historical data of TCM extraction process, the relevant knowledge of determining extraction parameters is mined, which is used to guide technologists to choose the influencing factors and the level of each factor in orthogonal test. Theoretical analysis and simulation research show that this method has fast learning speed, good tracking performance, strong generalization ability, low dependence on samples, and good popularization ability. In today's information explosion era, the combination of data mining technology and TCM is an inevitable trend. Data mining technology will further accelerate the pace of knowledge renewal of TCM, lay a solid foundation for the construction of modern TCM theory, and is the only way for the vigorous development of TCM.

## Data Availability

The data used to support the findings of this study are available from the corresponding author upon request.
